# Three Cases of Cerebral Meningitis

**Published:** 1897-12

**Authors:** Francis E. Fronczak

**Affiliations:** Visiting physician and surgeon to the Felician Sisters Institute; and to the Polish Orphan Asylum at Cheektowaga, N. Y.


### Clinical Report.

### THREE CASES OF CEREBRAL MENINGITIS.

By FRANCIS E. FRONCZAK, A. M., M. D.,

Visiting physician and surgeon to the Felician Sisters Institute ; and to the Polish Orphan Asylum at Cheektowaga, N. Y.

CASE I.—Early one morning I was asked to attend a dying child. Arriving at the bedside I found a six-year-old boy in an unconscious condition with rapid Cheyne-Stokes respiration, a pulse of 180 and temperature at 109°. I could not believe my thermometer at first, but as another physician, who was called by some other member of the family, arrived at this time, I asked him to take the temperature, and again 109° was registered. The case being now left in my hands I was at first at a loss what to do. The reduction of temperature, however, presented itself as most pressing, so I called for cold water and towels, applied a cold pack about the head and ordered an alcohol rub. The history of the case was short. Some time before the child had scarlatina, but got well and was in fairly good health until the day before I was called. At six in the evening he began to vomit and had a convulsion, then he complained of headache, and the lamplight pained his eyes. Later he became unconscious and had two or three involuntary evacuations. At 10 P. M. the boy felt better and was deeply asleep, being disturbed but once or twice during the night by a sharp pain in the head. At six in the morning he got up complaining of hunger. After partaking of some bread and milk he began to vomit again, this being followed by a convulsion and unconsciousness, in which condition I found him. His head was retracted, pupils contracted, fingers clinched over the thumb, and the legs drawn up. The child never regained consciousness, dying about noon-time the same day.

CASE II.—A week later I was called to see a woman who but a few hours before was in fairly good condition, and now was supposed to be dying. The patient, S., 60 years of age, was lying in a bed in a comatose condition, head drawn back, and pupils irregularly contracted. Respiration of the Cheyne-Stokes type, pulse irregular and rapid, temperature 97° F. I learned from the patient's daughter that she complained lately of loss of appetite, chills and fever, constipation, lateral headaches, especially of the left side and of earache. On examination I found purulent material in the left ear. The woman could not be aroused from her unconsciousness, stimulants brought no response, and shortly after my arrival she died.

CASE III.—Boy, age twelve, head bandaged, restless, now and then uttering a shrill cry. . History of the case was that he fell from a freight car and hurt his head. Arriving home his mother washed the wound with cold water, and buying five cents' worth of carbolated vaseline, applied the same on a dirty piece of linen and sent the boy to play. A few hours later he complained of headache, weakness and vomited. After being given some whisky he soon rallied, but would not eat his supper, saying he could not eat. About fifteen hours after the accident I was called, as he became restless and complained of headache. I cleaned the wound as best I could under the circumstances I had to contend with, the mother objecting to the removal of the surgically clean bandage (?) she applied to the wound earlier in the day. Three hours later I was summoned again, and found the boy in a semi-comatose condition, breathing rapidly, about sixty a minute; pulse was nearly 200; temperature, 103½° F.; pupils irregularly contracted and every touch seemed to pain him terribly. He had one or two convulsive spasms during my presence and died within a few minutes.

The question will now arise as to the nature of these three cases, similar to each other in some features, and again unlike in other characteristics. No doubt that they were cases of cerebral meningitis. The first most probably secondary to the scarlet fever from which the child suffered a short time before; the second case was a result of otitis media, from which the woman previously suffered. The third case of meningitis was traumatic in its origin. Each could have been prevented had it been properly and early attended. Each of these a gruesome and costly lesson that a trifling matter, to all appearances, may bring serious results. The temperature varying, as it did from 109° F. in the first case to 97° F. in the second, being another proof how greatly it may differ in the same disease; in one a hyperpyrexia, in the other case almost two degrees below normal.

508 Fillmore Avenue, Buffalo.

